# Psychosocial and clinical determinants of medication adherence among elderly chronic disease patients in China

**DOI:** 10.3389/fphar.2026.1836686

**Published:** 2026-06-08

**Authors:** Yujie Zhang, Yongli Han, Aning Sun, Mingfen Wu

**Affiliations:** 1 Department of Pharmacy, Beijing Tiantan Hospital, Capital Medical University, Beijing, China; 2 Department of Pharmacy, Linfen Central Hospital, Linfen, China

**Keywords:** chronic diseases, medication adherence, medication literacy, self-efficacy, social support

## Abstract

**Background:**

Identifying psychosocial factors associated with medication adherence is critical to optimize chronic disease management in elderly patients. This study aimed to explore key psychosocial and clinical determinants of medication adherence and their synergistic interactions among home-dwelling elderly patients with chronic diseases.

**Methods:**

A cross-sectional study was conducted among 952 elderly patients. Data were collected using validated scales including Adherence to Refills and Medications Scale (ARMS), Self-efficacy for Appropriate Medication Use Scale (SEAMS), Social Support Rate Scale (SSRS), Beliefs about Medical Questionnaire (BMQ), and The Adapted Chinese medication literacy measure (ChMLM). Univariate and multivariable logistic regression, together with interaction analysis, were used to explore associated factors and their synergistic effects.

**Results:**

Among 952 participants (mean age 70.32 ± 6.95 years, 51.47% male), multivariable analysis showed that living with a caregiver (OR = 6.91, 95% CI: 2.79–17.70, p < 0.001), higher self-efficacy (OR = 1.13, 95% CI: 1.10–1.17, p < 0.001), Support utilization (OR = 1.09, 95% CI: 1.01–1.17, p = 0.027), stronger medication necessity beliefs (OR = 1.06, 95% CI: 1.02–1.11, p = 0.005), and better medication proficiency (OR = 1.02, 95% CI: 1.00–1.04, p = 0.018) were independently associated with good adherence. Conversely, higher medication concerns (OR = 0.94, 95% CI: 0.91–0.98, p = 0.003), Objective support (OR = 0.91, 95% CI: 0.84–0.98, p = 0.020) and more medication knowledge (OR = 0.97, 95% CI: 0.96–0.99, p < 0.001) were associated with poorer adherence. Critically, Six significant synergistic interactions were identified, including Necessity × Concerns and Knowledge × Self-efficacy, suggesting mutual modification between these factors.

**Conclusion:**

Medication adherence is influenced by a complex network of determinants, where cognitive, behavioral, and social factors do not operate in isolation but interact synergistically. Multidimensional interventions targeting high-risk combinations, such as high medication knowledge with low self-efficacy, may effectively improve adherence.

## Highlights

- This study shifts focus from single-dimension to multi-factor interaction analysis in medication adherence research.
- Clarifies a key conditionality: high medication knowledge boosts adherence only when combined with high perceived necessity, self-efficacy, and residential support.
- Provides a comprehensive analytical framework integrating clinical, psychosocial, and contextual variables.
- The research results can provide evidence-based support for developing individualized chronic disease management and intervention measures.

## 1 Introduction

Chronic diseases, featuring long duration and insidious progression, are often incurable but controllable via sustained pharmacotherapy and lifestyle modification. Driven by ageing, unhealthy diets and sedentary lifestyles, their global prevalence has risen, posing a major public health challenge (NCD Risk Factor Collaboration NCD-RisC, 2019; Ye and Leeming, 2023). The 2017 Global Burden of Disease Study showed that non-communicable diseases (NCDs) caused 73.4% of global deaths, with cardiovascular disease, cancer, chronic respiratory disorders, and diabetes as the main causes (Roth et al., 2020; Bai et al., 2023).

Chronic diseases impose substantial physical, psychological and social burdens, requiring most patients to take lifelong medication. However, poor medication adherence is a critical barrier to management (Wong, 2020). A WHO survey indicated approximately 60% adherence in elderly chronic disease patients (Brown and Bussell, 2011), with 50% in developed countries (De Geest and Sabaté, 2003) and likely lower in developing countries. Poor adherence may worsen disease control, increase acute exacerbations, hospitalizations and costs (Wong, 2020), and prescription complexity negatively affects adherence (Burnier, 2024).

Non-adherence leads to adverse outcomes: Lloyd noted it burdens healthcare insurance (Lloyd et al., 2019); patients often readmit due to treatment discontinuation (Agana et al., 2019), and insufficient adherence correlates with higher cardiovascular events in hypertensive populations (Kim et al., 2016), highlighting its importance for chronic disease control.

Medication adherence is shaped by multidimensional factors, specifically the dynamic interplay of socioeconomic, treatment-related, disease-related, patient-related, and healthcare-system-related factors (Celebi et al., 2025; Schäfer, 2017), but most studies focus on single domains. Foreign studies often explore adherence factors based on theories, while domestic studies are mostly single-center, small-sample, lack psychosocial factors, and insufficiently explore interactive effects between factors, limiting the comprehensiveness of adherence research. Thus, a systematic investigation of six key dimensions (sociodemographic characteristics, clinical complexity, social support, self-efficacy, medication beliefs, medication literacy) is needed to quantify determinants’ influence.

The present study aims to identify psychosocial and clinical determinants of medication adherence in chronic disease patients, to advance understanding of adherence mechanisms, inform early-risk screening and targeted interventions, and ultimately improve therapeutic efficacy, healthcare utilisation and patient-reported quality of life.

## 2 Materials and methods

### 2.1 Study design and setting

This study was part of the National Natural Science Foundation of China (No. 72404196). A cross-sectional study was conducted in central China, with the survey carried out from January 1 to June 30, 2025. Using a simple sampling method, the sample size was determined based on a 90% power analysis. A total of 1007 patients participated in the questionnaire survey. This survey was conducted via face-to-face interviews, each lasting approximately 30 min. Given the large number of questions included in the questionnaire, pharmacists communicated with patients using an electronic questionnaire during the interviews and were responsible for recording the patients’ responses, so as to avoid respondent fatigue or incomplete answers. The overall length of the questionnaire was manageable for participants and did not have a negative impact on the completion rate. After strict data screening and quality control, 55 participants were excluded due to incomplete primary outcome dataand withdrawal of informed consent. Accordingly, the final valid sample size for analysis was 952. We further compared the baseline demographic and clinical characteristics between the excluded and retained groups. No statistically significant differences were observed between the two groups, as detailed in Supplementary Material 2, which indicated that sample loss did not introduce obvious selection bias.

### 2.2 Study participants

Inclusion criteria: Participants were eligible if they met the following conditions: (1) age ≥ 60 years; (2) diagnosed of at least one of the four chronic diseases—hypertension, type 2 diabetes, coronary heart disease, stroke; (3) currently receiving long-term pharmacological treatment; and (4) fully informed of the study objectives, willing to participate, and able to provide written informed consent.

Exclusion criteria: Patients were excluded if they had psychiatric disorders, malignant tumors, significant hearing impairment, or communication difficulties.

Recruitment and data collection: Convenience sampling was employed to recruit patients with chronic diseases from various communities. Pharmacists involved in the study completed two rounds of standardised training prior to data collection. Data were collected through structured face-to-face interviews: pharmacists administered the questionnaires verbally, recorded patients’ responses, and subsequently submitted the completed forms.

### 2.3 Instruments

The survey instrument developed for this study is available as Supplementary Material 1. The survey comprised three major sections: (1) Clinical factors: type of chronic disease (hypertension, dyslipidemia, diabetes, coronary heart disease, stroke, hyperuricemia or gout, asthma, atrial fibrillation, heart failure, and others), number of chronic conditions, disease duration, number of medications used, history of adverse drug reactions, and achievement of treatment targets. (2) Demographic factors: age, sex, marital status, smoking and alcohol use, living arrangement, employment status, educational level, monthly income, and method of medical payment. (3) Psychosocial factors: medication-related self-efficacy, medication beliefs, social support, and medication literacy.

Multiple validated scales were adopted for key variables, as follows:

Medication adherence: Measured using the sinicized *Adherence to Refills and Medications Scale* (ARMS) (Kripalani et al., 2009). Sinicized by two-way translation-back-translation, it is a self-reported scale. It showed good reliability (Cronbach’s α = 0.814 (Kripalani et al., 2009)) and content validity (verified by experts). This scale includes 12 items rated on a 4-point Likert scale (1 = never, 2 = sometimes, 3 = often, 4 = always). Lower total scores indicate better adherence. A score <16 was classified as good adherence, whereas a score ≥16 indicated poor adherence.

Self-efficacy: Assessed using the sinicized *Self-efficacy for Appropriate Medication Use Scale* (SEAMS) (Risser et al., 2007). Sinicized by the same method as ARMS, it is a self-reported scale. It had good reliability (Cronbach’s α = 0.89 (Risser et al., 2007)). The scale consists of 13 items, each rated on a 10-point Likert scale (1 = not at all confident, 10 = completely confident). Higher scores indicate greater self-efficacy in managing medication appropriately.

Social support: Evaluated with the sinicized Social Support Rating Scale (SSRS) (Xiao, 1994). A mature self-reported scale sinicized by standard translation- back- translation, it had good reliability (Cronbach’s α = 0.921 (Xiao, 1994)) The scale comprises 10 items across three dimensions: objective support (3 items), subjective support (4 items), and utilization of support (3 items). Items 1–4 and 8–10 are scored on a 4-point scale, whereas items 5–7 are rated according to the number of support sources reported. Total scores are classified as low (≤22), moderate (23–44), or high (45–66), with higher scores indicating stronger social support.

Medication Beliefs: Assessed with the sinicized *10-item Beliefs about Medicines Questionnaire* (BMQ) (Horne et al., 1999). This scale has shown good reliability in previous studies, with a reported Cronbach’s alpha coefficient of 0.738 (Horne et al., 1999). The instrument comprises two subscales: Necessity (6 items) and Concerns (6 items). Each item is rated on a 5-point Likert scale ranging from 1 (“strongly disagree”) to 5 (“strongly agree”). Subscale scores range from 5 to 25, with higher scores indicating stronger beliefs in the respective dimension. An overall medication-belief score was derived by subtracting the Concerns score from the Necessity score (range: −20 to +20). Negative values indicate negative beliefs about medication, whereas higher (more positive) scores indicate stronger positive beliefs.

Medication literacy: The questionnaire was adapted from the *Chinese Medication Literacy Measure* (ChMLM), with further testing for reliability and validity (Zhou et al., 2026). The adapted scale demonstrated excellent psychometric properties: Cronbach’s α = 0.920 (>0.80, indicating high internal consistency), Kaiser–Meyer–Olkin (KMO) value = 0.938 (>0.80), and Bartlett’s test of sphericity was highly significant (p < 0.001), confirming robust construct validity. The final KAP-based medication literacy scale consists of 55 items across three domains: medication-related knowledge, attitudes, and practices. Higher total scores represent better medication literacy.

### 2.4 Ethical approval and considerations

Prior to the study, written informed consent was obtained from all participants. The study was approved by the Ethics Committee of Beijing Tiantan Hospital, Capital Medical University (Approval No. KY 2024-408-02).

### 2.5 Statistical analysis

1. Descriptive statistical analysis: Basic descriptive statistics were used to summarize demographic and clinical characteristics.
2. Inferential statistical analysis: Between-group comparisons were performed using the Mann–Whitney U test, Fisher’s exact test, and χ^2^ test. A multivariable binary logistic regression model was constructed to identify independent predictors. The likelihood-ratio test was used for model goodness-of-fit evaluation. Results were reported as β, SE, OR, 95% CI, and two-tailed p-values, with p < 0.05 considered statistically significant.

## 3 Results

### 3.1 Basic characteristics of the participants

The analysis included 952 participants with a mean age of 70.32 ± 6.95 years. Half of the cohort (50.84%) were aged 60–69 years, and 51.47% were male. Most had an educational level of high school or below. Details characteristics are presented in Table 1. A total of 69.43% had two or more chronic conditions, and 49.79% had a disease duration of more than 10 years. Details characteristics are presented in Table 2.

**TABLE 1:** Demographic characteristics of the participants.

Characteristics	n	%	Characteristics	n	%
Gender	​	​	Educational level	​	​
Male	490	51.47	Illiterate	68	7.14
Female	462	48.53	Primary school	244	25.63
Age	​	​	Junior high schools	279	29.31
60–69	484	50.84	Senior high school	206	21.64
70–80	384	40.34	Junior college	114	11.97
>80	84	8.82	Undergraduate or above	41	4.31
Marital status	​	​	Smoking or not	​	​
Married	787	82.67	No smoking	659	69.22
Single	165	17.33	Smoking	220	23.11
Drinking or not	​	​	Quit smoking	73	7.67
No drinking	674	70.80	Monthly income (CNY)	​	​
Drinking	231	24.26	<1000	149	15.65
Quit drinking	47	4.94	1000 ~ 3000	223	23.42
Reimbursement type	​	​	3000–5000	331	34.77
Publicly-funded medical care	36	3.78	5000–10,000	225	23.63
Medical insurance for urban and rural residents or new rural cooperative medical system	414	43.49	>10,000	24	2.52
Self-funded	12	1.26	Living arrangement	​	​
Medical insurance for works	490	51.47	Living alone	111	11.66
Working status	​	​	With spouse	622	65.34
Taking care of grandchildren	133	13.97	Have a nanny/caregiver	41	4.31
Re-employment or other work	64	6.72	With children	178	18.70
Retired at home	755	79.31	​	​	​

CNY, Chinese Yuan (¥).

**TABLE 2:** Clinical characteristics of the participants.

Characteristics	N	%	Characteristics	n	%
Number of medications used	​	​	Disease duration	​	​
1–2	314	32.98	<1 year	31	3.26
3–5	453	47.58	1 ~ 5 years	203	21.32
>5	185	19.43	5 ~ 10 years	244	25.63
Chronic diseases	​	​	>10 years	474	49.79
Hypertension	779	81.83	Number of chronic conditions	​	​
Type 2 diabetes	395	41.49	1	291	30.57
Coronary heart disease	253	26.58	2	327	34.35
Stroke	156	16.39	3	198	20.80
Hyperlipidemia	276	28.99	4	84	8.82
Hyperuricemia or gout	60	6.30	5	42	4.41
Atrial fibrillation	21	2.21	6	10	1.05
Asthma	24	2.52	History of adverse drug reactions	​	​
Heart failure	14	1.47	Yes, but patient can’t describe the details clearly	102	10.71
Others	118	12.39	Yes, patient can describe the details clearly	109	11.45
​	​	​	Never occurred before	741	77.84

### 3.2 Univariate analysis

To identify factors associated with medication adherence among older adults with chronic diseases, univariate analyses were conducted on demographic, clinical, and psychological variables, and the results are presented in Tables 3, 4.

**TABLE 3:** Results of the chi-square test analysis of factors influencing medication adherence in patients with chronic diseases.

Variable	n	Medication adherence/n		χ^2^	P-value	Variable	n	Medication adherence/n		χ^2^	P-value
		Good	Poor					Good	Poor		
Gender	​	​	​	0.24	0.628	Number of chronic conditions	​	​	​	12.39	0.029
Male	490	224	266	​	​	1	291	152	139	​	​
Female	462	203	259	​	​	2	328	133	195	​	​
Age	​	​	​	0.73	0.695	3	198	78	120	​	​
60–69	484	220	264	​	​	4	83	40	43	​	​
70–80	384	173	211	​	​	5	42	21	21	​	​
>80	84	34	50	​	​	6	10	3	7	​	​
Marital status	​	​	​	0.0072	0.932	Disease duration	​	​	​	5.57	0.135
Married	787	352	435	​	​	<1 year	31	15	16	​	​
Single	165	75	90	​	​	1 ~ 5 years	203	80	123	​	​
Educational level	​	​	​	35.12	***	5 ~ 10 years	244	103	141	​	​
Illiterate	68	21	47	​	​	>10 years	474	229	245	​	​
Primary school	244	84	160	​	​	Number of medications used	​	​	​	5.76	0.056
Junior high school	279	121	158	​	​	1–2	314	155	159	​	​
Senior high school	206	111	95	​	​	3–5	453	201	252	​	​
Junior college	114	69	45	​	​	>5	185	71	114	​	​
Undergraduate or above	41	21	20	​	​	History of adverse drug reactions	​	​	​	7.49	0.024
Smoking or not	​	​	​	2.73	0.255	Yes, but the description was not clear	102	48	61	​	​
No smoking	659	301	358	​	​	Yes, and the patient can clearly describe	109	33	69	​	​
Smoking	220	100	120	​	​	No	741	346	395	​	​
Quit smoking	73	48	27	​	​	Level of social support	​	​	​	26.05	***
Drinking or not	​	​	​	0.21	0.900	Low level	18	9	9	​	​
No drinking	674	301	373	​	​	Moderate level	674	267	407	​	​
Drinking	231	106	125	​	​	High level	260	151	109	​	​
Quit drinking	47	20	27	​	​	Blood pressure controlled to target	​	​	​	16.03	***
Living arrangement	​	​	​	4.36	0.037	No	165	58	107	​	​
Living alone	111	39	72	​	​	Not monitored	25	6	19	​	​
Not alone	841	388	453	​	​	Yes	591	294	297	​	​
Working status	​	​	​	1.39	0.498	Blood lipids controlled to target	​	​	​	8.46	0.015
Taking care of grandchildren	133	57	76	​	​	No	139	48	91	​	​
Re-employment or other work	64	33	31	​	​	Not monitored	48	17	31	​	​
Retired at home	755	337	418	​	​	Yes	323	155	168	​	​
Monthly income (CNY)	​	​	​	26.78	***	Blood sugar controlled to target	​	​	​	10.83	**
<1000	149	56	93	​	​	No	176	60	116	​	​
1000 ~ 3000	223	81	142	​	​	Not monitored	21	8	13	​	​
3000 ~ 5000	331	146	185	​	​	Yes	201	102	99	​	​
5000 ~ 10,000	225	130	95	​	​	Whether gout flares up	​	​	​	0.38	0.827
>10,000	24	14	10	​	​	Uric acid is high, no gout attacks	13	6	7	​	​
Reimbursement type	​	​	​	7.46	0.059	No	36	15	21	​	​
Publicly-funded medical care	36	17	19	​	​	Yes	17	6	11	​	​
Medical insurance for urban and rural residents	414	171	243	​	​	Uric acid/Gout controlled to target	​	​	​	0.12	0.940
Self-funded	12	9	3	​	​	Not monitored	16	7	9	​	​
Medical insurance for workers	490	230	260	​	​	No	26	10	16	​	​
Chronic disease	​	​	​	10.60	0.304	Yes	24	10	14	​	​
Hypertension	779	357	422	​	​	Angina pectoris attacks	​	​	​	1.410	0.235
Diabetes	395	168	227	​	​	No	224	103	121	​	​
Coronary heart disease	253	113	140	​	​	Yes	28	9	19	​	​
Stroke	156	64	92	​	​	​	​	​	​	​	​
Hyperlipidemia	276	123	153	​	​	​	​	​	​	​	​
Others	237	91	146	​	​	​	​	​	​	​	​

CNY, Chinese Yuan (¥); χ^2^, chi-square statistic; p-value from Pearson’s chi-square test; ** indicates that the p-value is less than 0.01; *** indicates that the p-value is less than 0.001.

**TABLE 4:** Mann-Whitney U test analysis of influencing factors of medication adherence in patients with chronic diseases.

Scales	U	W	Z	p
BMI	109,500	−27003	−32.94	0.781
Social support	90,635	−47440	−37.81	***
Utilization of support	95,907	−42168	−36.56	***
Objective support	107,548.5	−30526.5	−33.8	0.271
Subjective support	91,066	−47009	−37.71	***
SEAM	57,871.5	−80203.5	−45.57	***
Medication beliefs	82,921	−55154	−39.64	***
Medication necessity	83,282	−54793	−39.55	***
Medication concerns	131,345	−6730	−28.16	***
Medication literacy	87,173	−50902	−38.63	***
Medication knowledge	105,990.5	−32084.5	−34.17	0.148
Medication attitude	87,632.5	−50442.5	−38.52	***
Medication practice	78,210	−59865	−40.75	***

*** indicates that the p-value is less than 0.001; Abbreviations: U, Mann–Whitney U-test; W, wilcoxon sum of ranks; Z, Standardized test statistic for Wilcoxon signed-rank test; p, level of significance; SEAM, Self-efficacy for Appropriate Medication.

Among sociodemographic factors, education (χ^2^ = 35.12, p < 0.001), living arrangement (χ^2^ = 4.36, p = 0.037), and monthly income (χ^2^ = 26.78, p < 0.001) were significantly associated with medication adherence. No significant associations were found for gender, age, marital status, employment, smoking, alcohol use, or medical insurance type.

For clinical factors, the number of chronic conditions (χ^2^ = 12.39, p = 0.029), as well as blood pressure (χ^2^ = 16.03, p < 0.001), lipid (χ^2^ = 8.46, p = 0.015), and glucose control (χ^2^ = 10.83, p = 0.004), were significantly related to adherence. Patients with a history of adverse drug reactions also showed better adherence (χ^2^ = 7.49, p = 0.024). In contrast, specific disease type, disease duration, and the number of medications were not significant.

For psychosocial factors, social support (χ^2^ = 26.05, p < 0.001), medication self-efficacy (U = 57,871.5, Z = −45.57, p < 0.001), medication beliefs (U = 82,921, Z = −39.64, p < 0.001), and medication literacy (U = 87,173, Z = −38.63, p < 0.001) were all significantly associated with adherence. Subgroup analyses indicated significant differences in subjective support, support utilization, medication necessity, attitudes, and skills, while objective support and medication knowledge showed no significant differences.

### 3.3 Multivariate analysis

All variables that were significant in the univariate analysis were included in a multivariable logistic regression model. After adjustment for potential confounders, several factors remained independently associated with medication adherence. The results are represented in Table 5 **.**

**TABLE 5:** Binary logistic regression analysis of factors influencing medication adherence.

Variable	β	SE	OR	95%CI	P
(Intercept)	−3.95	1.03	0.02	(0.00–0.14)	<0.001
Living arrangement (ref = living alone)					
Have a nanny/caregiver	1.93	0.47	6.91	(2.79–17.70)	<0.001
With spouse	0.67	0.28	1.96	(1.13–3.42)	0.017
With children	1.13	0.32	3.08	(1.67–5.76)	<0.001
Education (ref = undergraduate or above)					
Illiterate	−0.28	0.55	0.75	(0.25–2.21)	0.607
Primary school	−0.16	0.48	0.85	(0.33–2.17)	0.738
Junior high school	0.17	0.46	1.18	(0.48–2.91)	0.719
Senior high school	0.49	0.46	1.64	(0.66–4.07)	0.288
Junior college	0.59	0.47	1.80	(0.71–4.55)	0.213
Monthly income (ref = >10,000 CNY)					
<1000	0.00	0.61	1.00	(0.30–3.25)	0.994
1000–3000	−0.32	0.59	0.73	(0.23–2.27)	0.590
3000–5000	−0.45	0.56	0.64	(0.21–1.88)	0.422
5000–10,000	−0.31	0.55	0.73	(0.24–2.13)	0.575
Adverse drug reactions (ref = no)					
Yes, clearly described	−0.14	0.25	0.87	(0.53–1.42)	0.570
Yes, unclear description	−0.39	0.27	0.68	(0.40–1.15)	0.153
Number of chronic diseases (ref = 1)					
2	−0.61	0.19	0.55	(0.37–0.80)	0.002
3	−0.75	0.23	0.47	(0.30–0.73)	0.001
4	−0.39	0.30	0.68	(0.37–1.23)	0.206
5	−0.10	0.40	0.90	(0.41–1.99)	0.802
6	−1.44	0.85	0.24	(0.04–1.16)	0.090
Psychosocial factors					
SEAM	0.13	0.01	1.13	(1.10–1.17)	<0.001
Objective support	−0.10	0.04	0.91	(0.84–0.98)	0.020
Subjective support	0.02	0.02	1.02	(0.99–1.06)	0.231
Support utilization	0.09	0.04	1.09	(1.01–1.17)	0.027
Medication necessity	0.06	0.02	1.06	(1.02–1.11)	0.005
Medication concerns	−0.06	0.02	0.94	(0.91–0.98)	0.003
Medication knowledge	−0.03	0.01	0.97	(0.96–0.99)	<0.001
Medication attitude	0.02	0.02	1.02	(0.99–1.06)	0.221
Medication proficiency	0.02	0.01	1.02	(1.00–1.04)	0.018

Abbreviations: β, regression coefficient; SE, standard error; OR, odds ratio; 95% CI, calculated by the Wald method; p, level of significance; SEAM, Self-efficacy for Appropriate Medication. All models used binary logistic regression with AMRS, as the dependent variable (binary outcome).

As shown in Table 5, living arrangement was a strong predictor. Compared with patients living alone, those with a caregiver or nanny had the highest likelihood of good adherence (OR = 6.91, 95% CI: 2.79–17.70, p < 0.001). Patients living with children also showed significantly better adherence (OR = 3.08, 95% CI: 1.67–5.76, p < 0.001), as did those living with a partner (OR = 1.96, 95% CI: 1.13–3.42, p = 0.017). These results suggest that family or caregiver support plays a crucial role in facilitating adherence.

Medication self-efficacy (SEAMS score) was another strong determinant: each one-point increase in SEAMS was associated with a 13.4% increase in the probability of good adherence (OR = 1.13, 95% CI: 1.10–1.17, p < 0.001), underscoring the importance of patients’ confidence in managing their medication.

Social support also influenced adherence. Greater utilization of social support increased the likelihood of good adherence (OR = 1.09, 95% CI: 1.01–1.17, p = 0.027). Interestingly, higher objective support was inversely associated with adherence (OR = 0.91, 95% CI: 0.84–0.98, p = 0.020), suggesting that tangible support without effective utilization may not improve, and could even reduce, adherence.

Medication beliefs showed divergent effects: stronger necessity beliefs promoted adherence (OR = 1.06, 95% CI: 1.02–1.11, p = 0.005), whereas greater concerns about medications predicted poorer adherence (OR = 0.94, 95% CI: 0.91–0.98, p = 0.003).

Medication literacy was also independently associated. Knowledge scores were negatively related to adherence (OR = 0.97, 95% CI: 0.96–0.99, p < 0.001), while proficiency scores were positively related (OR = 1.02, 95% CI: 1.00–1.04, p = 0.018). This counterintuitive finding might suggest that knowledge alone, without the corresponding positive beliefs or self-efficacy, could potentially lead to increased awareness of side effects or treatment complexities, thereby hindering adherence. This underscores that practical ability and confidence in medication management may be more critical determinants than knowledge in isolation.

Other variables, including education level, income, adverse drug reactions, subjective support, and medication attitude, were not significant in the multivariate model. The overall model demonstrated good fit and discriminatory ability, with an AUC of 0.801, indicating strong predictive performance in distinguishing patients with good versus poor adherence.

### 3.4 Interaction effects

**FIGURE 1:**
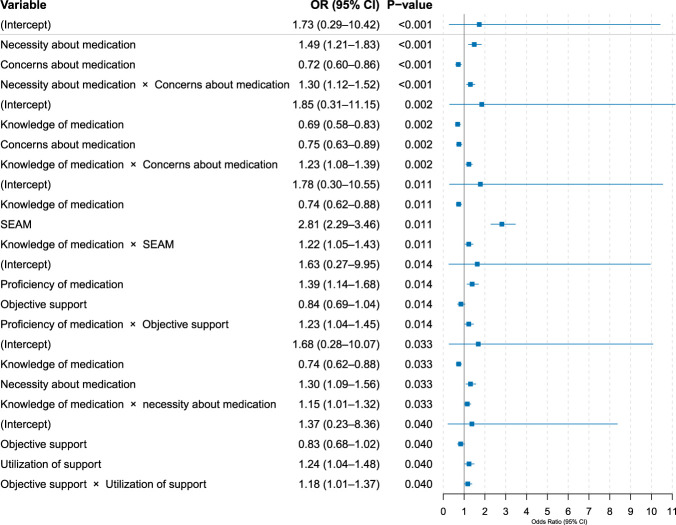
Forest plot of the multiplicative interaction affecting medication adherence in patients with chronic diseases. Abbreviations: OR, odds ratio; CI, confidence interval; P-value, likelihood-ratio test P-value for interaction; SEAM, Self-efficacy for Appropriate Medication.

Interaction effects were examined in Model 1 (unadjusted) and after full adjustment for all covariates listed in Table 6. Six multiplicative interaction terms remained significant after adjustment, indicating that their effects on adherence are mutually amplifying rather than simply additive (Figure 1).

**TABLE 6:** Multiplicative interactions affecting patients’ medication adherence.

Interaction	Model 1			Model 2 (adjusted*)		
	β	OR (95%CI)	P (LRT)	β	OR (95%CI)	P (LRT)
(Intercept)	−0.652	0.52 (0.30,0.90)	0.0001	0.547	1.73 (0.29,10.42)	<0.001
Necessity about medication	0.366	1.44 (1.19,1.75)	0.0001	0.396	1.49 (1.21,1.83)	<0.001
Concerns about medication	−0.31	0.73 (0.62,0.870)	0.0001	−0.332	0.72 (0.60,0.86)	<0.001
Necessity about medication × Concerns about medication	0.265	1.30 (1.13,1.50)	0.0001	0.266	1.30 (1.12,1.52)	<0.001
(Intercept)	−0.609	0.54 (0.32,0.94)	0.0004	0.613	1.85 (0.31,11.15)	0.002
Knowledge of medication	−0.357	0.7 (0.59,0.83)	0.0004	−0.368	0.69 (0.58,0.83)	0.002
Concerns about medication	−0.26	0.77 (0.66,0.91)	0.0004	−0.29	0.75 (0.63,0.89)	0.002
Knowledge of medication × Concerns about medication	0.208	1.23 (1.10,1.38)	0.0004	0.204	1.23 (1.08,1.39)	0.002
(Intercept)	−0.638	0.53 (0.31,0.91)	0.0133	0.578	1.78 (0.30,10.55)	0.011
Knowledge of medication	−0.295	0.74 (0.63,0.88)	0.0133	−0.304	0.74 (0.62,0.88)	0.011
SEAM	0.976	2.65 (2.19,3.21)	0.0133	1.035	2.81 (2.29,3.46)	0.011
Knowledge of medication × SEAM	0.179	1.20 (1.04,1.38)	0.0133	0.202	1.22 (1.05,1.43)	0.011
(Intercept)	−0.554	0.58 (0.33,0.99)	0.0128	0.488	1.63 (0.27,9.95)	0.014
Proficiency of medication	0.334	1.40 (1.17,1.67)	0.0128	0.328	1.39 (1.14,1.69)	0.014
Objective support	−0.16	0.852 (0.71,1.03)	0.0128	−0.168	0.85 (0.69,1.04)	0.014
Proficiency of medication × Objective support	0.196	1.22 (1.04,1.42)	0.0128	0.204	1.23 (1.04,1.45)	0.014
(Intercept)	−0.666	0.52 (0.30,0.89)	0.0111	0.518	1.68 (0.28,10.07)	0.033
Knowledge of medication	−0.291	0.75 (0.64,0.88)	0.0111	−0.304	0.74 (0.62,0.88)	0.033
Necessity about medication	0.241	1.27 (1.07,1.51)	0.0111	0.266	1.31 (1.09,1.56)	0.033
Knowledge of medication × Necessity about medication	0.154	1.17 (1.03,1.32)	0.0111	0.143	1.15 (1.01,1.32)	0.033
(Intercept)	−0.754	0.47 (0.27,0.82)	0.0178	0.317	1.37 (0.23,8.36)	0.040
Objective support	−0.175	0.84 (0.70,1.01)	0.0178	−0.183	0.83 (0.68,1.02)	0.040
Utilization of support	0.255	1.29 (1.10,1.52)	0.0178	0.214	1.24 (1.04,1.48)	0.040
Objective support × Utilization of support	0.175	1.19 (1.03,1.38)	0.0178	0.162	1.18 (1.01,1.37)	0.040

*Model 2 adjusted for sex, age, marital status, education, income, reimbursement type, living arrangement, chronic disease type, and other psychosocial covariates. β denotes the interaction coefficient; OR, odds ratio; CI, confidence interval; p (LRT), likelihood-ratio test P-value for interaction.

Interaction analyses were conducted to explore whether combinations of psychosocial and literacy factors exerted synergistic or antagonistic effects on adherence. Six interaction terms remained significant after adjusting for sociodemographic and clinical confounders.

Medication necessity × Concerns: Patients with strong necessity beliefs but also high concerns exhibited markedly different adherence patterns (OR = 1.30, 95% CI: 1.12–1.52, p < 0.001). This suggests that the positive effect of necessity beliefs can be offset or amplified depending on the level of concern.

Medication knowledge × Concerns: The combination of higher knowledge and higher concerns was associated with poorer adherence (OR = 1.23, 95% CI: 1.08–1.39, p = 0.002), indicating that knowledge alone may heighten awareness of side effects and thereby reduce adherence if not accompanied by positive beliefs.

Medication knowledge × Self-efficacy: Patients with both greater medication knowledge and stronger self-efficacy showed better adherence (OR = 1.22, 95% CI: 1.05–1.43, p = 0.011). This demonstrates a synergistic effect, where confidence in medication management enables patients to effectively apply their knowledge.

Medication proficiency × Objective support: Higher medication proficiency combined with higher objective support significantly improved adherence (OR = 1.23, 95% CI: 1.04–1.45, p = 0.014). This suggests that practical skills and tangible resources reinforce each other in supporting adherence.

Medication knowledge × Necessity: The interaction between knowledge and necessity beliefs was also significant (OR = 1.15, 95% CI: 1.01–1.32, p = 0.033). Patients who not only understood their medications but also perceived them as necessary were more likely to adhere.

Objective support × Support utilization: Although objective support alone was negatively associated with adherence, its combination with higher utilization of support resulted in improved adherence (OR = 1.18, 95% CI: 1.01–1.37, p = 0.040). This highlights the importance of effectively mobilizing available resources.

Taken together, these findings demonstrate that medication adherence is shaped not only by individual predictors but also by the interplay between cognitive, behavioral, and social factors. Synergistic effects between knowledge, beliefs, skills, and support underscore the need for multifaceted interventions targeting multiple dimensions simultaneously.

## 4 Discussion

This study systematically investigated psychosocial and clinical determinants of medication adherence among elderly patients with chronic diseases. By combining univariate, multivariable, and interaction analyses, we demonstrated that adherence is influenced by a complex interplay of sociodemographic, clinical, cognitive, and social factors, rather than by any single determinant. Importantly, the interaction analysis revealed synergistic and antagonistic effects, underscoring the need to consider multidimensional pathways when designing interventions.

### 4.1 Sociodemographic and clinical factors

Our findings confirmed that education, income, and living arrangement significantly affect adherence, consistent with earlier studies in elderly populations with diabetes and hypertension (Chen et al., 2024; Almaghamsi and Alzahrani, 2024; Khojah et al., 2024). Higher education and income likely facilitate access to health information and medical services, thereby improving medication proficiency and reducing economic barriers (Qin et al., 2024; Shen et al., 2020; Teppo et al., 2022). Living alone was consistently identified as a risk factor for poor adherence (Lee and Jeong, 2025; Lee et al., 2022), echoing prior cohort evidence that household companionship fosters better treatment continuity.

Clinically, adherence was associated with the number of chronic diseases, history of adverse drug reactions, and achievement of treatment targets (blood pressure, lipid, and glucose control). These results indicate a bidirectional relationship: patients who adhere more closely are better able to control disease, while improved health outcomes may, in turn, reinforce adherence (Song et al., 2024; Aravindakshan et al., 2021; Katzmann et al., 2018; Reach et al., 2019). However, comorbidities and adverse reactions can complicate regimens, induce anxiety, and ultimately discourage adherence (Abegaz et al., 2017; Margolis et al., 2025).

### 4.2 Psychosocial determinants

Social support, medication self-efficacy, medication beliefs, and medication literacy emerged as key psychosocial predictors (Guo et al., 2023; Yu et al., 2024; Upamali and Rathnayake, 2023; Sawalmeh et al., 2025; Liu et al., 2025; Kara, 2022; Shen et al., 2020). Stronger social support and higher self-efficacy consistently improved adherence, aligning with prior findings in stroke,Dialysis and myasthenia gravis populations (Saguban et al., 2025; Turan et al., 2019; Yu et al., 2024). However, our multivariable results showed a nuanced picture: objective support alone was negatively correlated with adherence, while support utilization exerted a positive effect. This suggests that resources without active engagement may foster dependency, whereas patients who effectively mobilize support benefit from reinforcement loops of reminders, feedback, and encouragement.

Medication beliefs within the necessity–concern framework were also pivotal (Shahin et al., 2021; Shahin et al., 2020). As in previous research (Nugraheni et al., 2020; Te Paske et al., 2023; Qiao et al., 2020; Sipos et al., 2021), necessity beliefs promoted adherence while concerns impeded it. Importantly, we found a significant multiplicative interaction: strong necessity beliefs attenuated the negative influence of concerns, highlighting the dynamic balance between perceived benefits and fears. This insight provides a basis for tailored counseling: patients with high concerns require both empathic acknowledgment and reinforcement of necessity, whereas those with low concerns may benefit primarily from regimen simplification.

Medication literacy further influenced adherence (Plaza-Zamora et al., 2020; Yang et al., 2025; Ku and Kegels, 2015), but with a paradoxical pattern. Knowledge alone was negatively associated with adherence in the multivariate model. We hypothesize that this could occur if awareness of potential side effects or treatment complexities, in the absence of strong necessity beliefs or adequate support, inadvertently amplifies patient anxiety or concerns—a phenomenon that could be described as “knowing but not believing,” or even “knowing too much without adequate support.” However, when combined with high necessity beliefs, self-efficacy, or support utilization, knowledge became a facilitator of adherence. This highlights that knowledge must be contextualized within a supportive cognitive and behavioral framework to yield positive effects.

### 4.3 Interaction mechanisms and implications

Six significant interactions—necessity × concern, knowledge × concern, knowledge × self-efficacy, proficiency × objective support, knowledge × necessity, and objective support × utilization—demonstrated that adherence is shaped by synergistic mechanisms rather than isolated factors. These interactions carry practical implications: ① This study found that the effect of knowledge is moderated by medication beliefs. Firstly, the perception of medication necessity is a prerequisite for knowledge to exert a positive effect: knowledge promotes medication adherence only when patients recognize the need for medication; if patients deny this necessity, knowledge may instead strengthen their resistance by deepening awareness of medication limitations. Secondly, the level of medication concerns determines the direction of the effect of knowledge. For patients who recognize the necessity of medication but have high concerns, knowledge can reduce such concerns by clarifying misunderstandings and answering questions, thereby improving medication adherence. This reflects the differential value of knowledge under different states of medication beliefs. ② Objective support × Support utilization: Objective support refers to the actual spiritual or material support obtained by an individual, including direct material assistance, as well as the tangible existence and involvement of social relations. It is an objectively existing reality. Support utilization describes the extent to which an individual makes use of the available social support. Some people are able to access support resources but refuse to accept help from others. Objective support improves adherence only when patients actively engage with available resources, underscoring the need for interventions that strengthen patients’ help-seeking and utilization skills. ③Self-efficacy as a moderator: High self-efficacy enables patients to transform knowledge into effective practices, emphasizing the importance of confidence-building strategies in patient education.

### 4.4 Practical and policy implications

Our findings highlight the importance of multidimensional, interaction-oriented interventions. Pharmacist-led education should go beyond improving knowledge to also strengthen necessity beliefs, alleviate concerns, and enhance self-efficacy. Our findings suggest that interventions might need to prioritize patients who have adequate knowledge but low positive beliefs or poor social support, as this subgroup appeared to be at a higher risk of poor adherence in our study. For solitary or low-income patients, building utilization skills and mobilizing community or family support may be as critical as financial subsidies.

## 5 Conclusion

This cross-sectional study identified multiple factors and their interactions that are associated with medication adherence in elderly patients with chronic diseases. Medication beliefs, literacy, social support, and self-efficacy are pivotal, but their effects are context-dependent and mutually reinforcing. Interventions that integrate cognitive, behavioral, and social components—particularly those addressing medication beliefs, self-efficacy, and support utilization—hold promise for improving adherence. However, longitudinal or interventional studies are needed to confirm the causal nature of these relationships and the efficacy of such multifaceted interventions.

## 6 Strengths and implications

This study incorporates biomedical, psychological and social context variables simultaneously, providing a relatively comprehensive depiction of adherence determinants. By introducing multiplicative interaction analysis innovatively into research on medication adherence among elderly chronic patients, we clarified that high knowledge translates into high adherence only when perceived necessity, self-efficacy and residential support are all high. Health professionals should supplement routine medication literacy education with self-efficacy training, and develop parallel motivation–concern interventions for the ‘high knowledge–high concern–low support’ subgroup. This would tangibly improve adherence and ease the burden of chronic disease management.

## 7 Limitations

This study has several limitations that should be considered when interpreting the results. First and foremost, the cross-sectional design precludes the establishment of causal relationships between the identified factors and medication adherence. The associations observed are simultaneous, and the direction of influence in some cases (e.g., between adherence and achieving treatment targets) could be bidirectional. Second, medication adherence and several key psychosocial variables (e.g., self-efficacy, beliefs) were assessed using self-reported measures, which are susceptible to social desirability and recall biases. The use of objective adherence measures (e.g., pill counts, pharmacy refill data) in future research would strengthen the findings. Third, the study participants were recruited from specific communities in central China using convenience sampling, which may limit the generalizability of our findings to other populations or healthcare settings. Fourth, despite adjusting for a wide range of potential confounders in our multivariate models, the possibility of residual confounding due to unmeasured variables (e.g., cognitive function, healthcare system factors) cannot be entirely ruled out.

## Data Availability

The raw data supporting the conclusions of this article will be made available by the authors, without undue reservation.
