# Supplementary material for: Psychosocial and clinical determinants of medication adherence among elderly chronic disease patients in China
Source: Front Pharmacol. 2026 Jun 8;17:1836686. doi: 10.3389/fphar.2026.1836686 (PMC13284060; doi:10.3389/fphar.2026.1836686)
Supplement: Supplementary file 1 [file Supplementaryfile1.docx]

**Survey on Home Medication Adherence in Elderly Patients with Chronic Diseases**

Dear Pharmacist,

Thank you for participating in the research on home medication adherence among elderly patients with chronic diseases. This survey is funded by the National Natural Science Foundation of China (Grant No. 72404196). This survey aims to understand the psychosocial factors influencing home medication adherence in elderly patients with chronic diseases in China. The survey targets patients aged 60 and above, diagnosed with at least one of the following four chronic diseases: hypertension, diabetes, coronary heart disease, or stroke, and who are on long-term medication therapy. This survey must be conducted face-to-face by a pharmacist, who will complete the questionnaire online.

**I. Pharmacist Information**

1. Name:
2. Province:
3. Institution:
4. Phone Number:

**II. Patient Basic Information**

1. **Gender:** ① Male □; ② Female □;
2. **Age:** years old;
3. **Height:** meters;
4. **Weight:** Kg;
5. **Marital Status:** ① Unmarried □; ② Married □; ③ Divorced □; ④ Widowed □;
6. **Education Level:** ① Illiterate □; ② Primary School □; ③ Junior High School □; ④ Senior High School □; ⑤ College/Technical Secondary School □; ⑥ Bachelor's Degree □; ⑦ Master's Degree or above □
7. **Do you smoke?** ① Non-smoker □; ② Smoker □; ③ Quit smoking □
8. **Do you drink alcohol?** ① Non-drinker □; ② Drinker □; ③ Quit drinking □
9. **Living Situation:** ① Live alone □; ② With spouse □; ③ Live with children □; ④ With a caregiver/other □
10. **Current Work Status:** ① Retired □; ② Re-employed/Other work □; ③ Caring for grandchildren □
11. **Monthly Income:** ① < 1000 RMB □; ② < 3000 RMB □; ③ 3000 ~ 5000 RMB □; ④ 5000 ~ 10000 RMB □; ⑤ > 10,000 RMB □
12. **Primary Healthcare Payment Method:** ① Out-of-pocket □; ② Employee Basic Medical Insurance □; ③ Government Insurance □; ④ Urban and Rural Resident Basic Medical Insurance/New Rural Cooperative Medical Scheme □; ⑤ Commercial Insurance □;
13. **Which chronic diseases do you have? (Multiple choices allowed)** ① Hypertension □; ② Diabetes □; ③ Coronary Heart Disease □; ④ Stroke □; ⑤ Hyperlipidemia □; ⑥ Hyperuricemia/Gout □; ⑦ Atrial Fibrillation □; ⑧ Asthma □; ⑨ Heart Failure □; ⑩ Other □
14. **Total number of chronic diseases:** types.
15. **Duration of Chronic Disease(s):** ① < 1 year □; ② 1 ~ 5 years □; ③ 5 ~ 10 years □; ④ > 10 years □;
16. **Current number of medications taken** (including prescription and over-the-counter drugs): types.
17. **Have you experienced any adverse drug reactions?** ① Yes, and the patient can describe clearly □; ② Yes, but the description is unclear □; ③ No □
18. **Please report the most recent monitoring results and treatment goal achievement based on the patient's conditions:**

**(1) (For patients with hypertension)** ① Systolic Blood Pressure mmHg; ② Diastolic Blood Pressure mmHg. Has blood pressure been controlled to target in the last 3 months? Target achieved □; Not achieved □; Not monitored □.
**(2) (For patients with diabetes)** ① Fasting Blood Glucose mmol/l; ② 2-hour Postprandial Blood Glucose mmol/l; ③ Glycated Hemoglobin (HbA1c) %. Has blood glucose been controlled to target in the last 3 months? Target achieved □; Not achieved □; Not monitored □.
**(3) (For patients with hyperlipidemia)** ① Total Cholesterol (TC) mmol/l; ② Low-Density Lipoprotein Cholesterol (LDL-C) mmol/l; ③ Triglycerides (TG)

mmol/l. Have lipids been controlled to target in the last 3 months? Target achieved □; Not achieved □; Not monitored □.
**(4) (For patients with hyperuricemia/gout)** ① Serum Uric Acid Level μmol/l; ② Has the patient had any gout attacks in the last 6 months? ① No; ② Yes, please specify number of attacks times; ③ Only high uric acid, no gout. Have uric acid levels/gout been controlled to target in the last 3 months? Target achieved □; Not achieved □; Not monitored □.
**(5) (For patients with coronary heart disease)** ① Have you had any angina attacks in the last 6 months? ① No; ② Yes, please specify number of attacks times;

**III. Medication Adherence Rating Scale (MARS)**

| No. | Item | Frequency of Occurrence | | | |
| --- | --- | --- | --- | --- | --- |
|  |  | Never | Sometimes | Often | Always |
| 1 | Do you ever forget to take your medicine? | 1 | 2 | 3 | 4 |
| 2 | Do you ever decide not to take your medicine? | 1 | 2 | 3 | 4 |
| 3 | Do you ever forget to get your prescription refilled? | 1 | 2 | 3 | 4 |
| 4 | Do you ever run out of your medicine? | 1 | 2 | 3 | 4 |
| 5 | Do you ever skip a dose of your medicine before a doctor's appointment? | 1 | 2 | 3 | 4 |
| 6 | Do you ever take less medicine than prescribed when you feel your symptoms are under control? | 1 | 2 | 3 | 4 |
| 7 | Do you ever skip a dose of your medicine if you feel unwell? | 1 | 2 | 3 | 4 |
| 8 | Do you ever neglect to take your medicine on time due to carelessness? | 1 | 2 | 3 | 4 |
| 9 | Do you ever adjust your medication dose (e.g., reduce or increase) based on your own judgment? | 1 | 2 | 3 | 4 |
| 10 | Do you ever forget to take your medicine if it needs to be taken more than once a day? | 1 | 2 | 3 | 4 |
| 11 | Do you ever delay getting your prescription refilled because of the cost? | 1 | 2 | 3 | 4 |
| 12 | Do you often plan ahead and refill your prescription before running out? (Reverse-scored) | 4 | 3 | 2 | 1 |

**IV. Beliefs about Medicines Questionnaire (BMQ)**

| **No.** | **Beliefs about Medicines** | **Strongly Agree** | **Agree** | **Uncertain** | **Disagree** | **Strongly Disagree** |
| --- | --- | --- | --- | --- | --- | --- |
| 1 | My current health depends on my medicines. | 5 | 4 | 3 | 2 | 1 |
| 2 | My quality of life would decrease if I stopped my medicines. | 5 | 4 | 3 | 2 | 1 |
| 3 | My condition would get worse if I stopped my medicines. | 5 | 4 | 3 | 2 | 1 |
| 4 | My future health depends on my medicines. | 5 | 4 | 3 | 2 | 1 |
| 5 | Taking my medicines prevents my condition from worsening. | 5 | 4 | 3 | 2 | 1 |
| 6 | I am worried about having to take chronic disease medicines. | 5 | 4 | 3 | 2 | 1 |
| 7 | I sometimes worry about the long-term effects of my medicines. | 5 | 4 | 3 | 2 | 1 |
| 8 | I do not understand the purpose of my medicines. | 5 | 4 | 3 | 2 | 1 |
| 9 | Taking medicines disrupts my life. | 5 | 4 | 3 | 2 | 1 |
| 10 | I sometimes worry about becoming too dependent on medicines. | 5 | 4 | 3 | 2 | 1 |

**V. Self-Efficacy for Appropriate Medication Use Scale (SEAMS)**

| **No.** | **How confident are you about taking your medications correctly under the following circumstances?** | **Not Confident (1 point)** | **Somewhat Confident (2 points)** | **Very Confident (3 points)** |
| --- | --- | --- | --- | --- |
| 1 | When your doctor changes your medicine? |  |  |  |
| 2 | When you find the newly purchased medicine has different instructions from the previous one? |  |  |  |
| 3 | When you need to take several different kinds of medicines each day? |  |  |  |
| 4 | When you need to take medicine more than once a day? |  |  |  |
| 5 | When you are traveling? |  |  |  |
| 6 | When you are very busy one day? |  |  |  |
| 7 | When your medicine causes side effects? |  |  |  |
| 8 | When there is no one to remind you? |  |  |  |
| 9 | When taking your medicine is inconvenient or troublesome? |  |  |  |
| 10 | When your daily routine gets disrupted? |  |  |  |
| 11 | When you are not sure about how to take your medicine? |  |  |  |
| 12 | When you are not sure about the time to take your medicine? |  |  |  |
| 13 | When you have other illnesses (like a cold or flu)? |  |  |  |

**VI. Social Support Rating Scale (SSRS)**

| **No.** | **Item Content** | | **Options** | | | |
| --- | --- | --- | --- | --- | --- | --- |
| 1 | How many close friends do you have whom you can get support and help from? | | (1) None; (2) 1-2; (3) 3-5; (4) 6 or more | | | |
| 2 | In the past year, you have been: | | (1) Living away from family, alone; (2) Frequently changing residence, mostly living with strangers; (3) Living with classmates/colleagues/friends; (4) Living with family | | | |
| 3 | You and your neighbors: | | (1) Never care about each other, just nod hello; (2) Might show slight care if encountering difficulties; (3) Some neighbors care about you; (4) Most neighbors care about you | | | |
| 4 | You and your colleagues: | | (1) Never care about each other, just nod hello; (2) Might show slight care if encountering difficulties; (3) Some colleagues care about you; (4) Most colleagues care about you | | | |
| 5 | Support and care from family members: *(Score for each category: 1-4)* |  | None | Minimal | Moderate | Full Support |
|  |  | Spouse (lover) |  |  |  |  |
|  |  | Parents |  |  |  |  |
|  |  | Children |  |  |  |  |
|  |  | Siblings |  |  |  |  |
|  |  | Other members |  |  |  |  |
| 6 | In the past, when you encountered emergency situations, what were the sources of financial support and practical help you received? | | (1) No source; (2) The following sources (multiple choices allowed): A. Spouse; B. Other family members; C. Relatives; D. Colleagues; E. Work unit; F. Official or semi-official organizations (Party, Youth League, Union, etc.); G. Non-governmental organizations (religious, social groups, etc.); H. Other (please list) _______ | | | |
| 7 | In the past, when you encountered emergency situations, what were the sources of comfort and concern you received? | | (1) No source; (2) The following sources (multiple choices allowed): A. Spouse; B. Other family members; C. Relatives; D. Colleagues; E. Work unit; F. Official or semi-official organizations (Party, Youth League, Union, etc.); G. Non-governmental organizations (religious, social groups, etc.); H. Other (please list) _______ | | | |
| 8 | How do you confide when you have worries? | | (1) Never confide in anyone; (2) Only talk to 1-2 very close individuals; (3) Will tell if friends ask proactively; (4) Actively talk about your worries to get support and understanding | | | |
| 9 | How do you seek help when you have worries? | | (1) Rely only on yourself, do not seek help from others; (2) Rarely ask others for help; (3) Sometimes ask others for help; (4) Often ask family, relatives, friends, or organizations for help when in difficulty | | | |
| 10 | Regarding group activities (e.g., Party organization, religious organization, labor union, etc.), you: | | (1) Never participate; (2) Sometimes participate; (3) Often participate; (4) Actively participate and engage enthusiastically | | | |

**VII. Survey on Scientific Medication Literacy and Knowledge for the Elderly**

| **No** | **Knowledge/Attitude Items** | **Strongly Agree** | **Agree** | **Neutral/Don't Know** | **Disagree** | **Strongly Disagree** |
| --- | --- | --- | --- | --- | --- | --- |
| 1 | When sick, one should try to get injections or IV infusions. | 1 | 2 | 3 | 4 | 5 |
| 2 | Injectable medications (shots, IVs) are safer than oral medications. | 1 | 2 | 3 | 4 | 5 |
| 3 | More expensive medicines are safer. | 1 | 2 | 3 | 4 | 5 |
| 4 | More expensive medicines are more effective. | 1 | 2 | 3 | 4 | 5 |
| 5 | When buying medicine, judge its quality by its brand and reputation. | 1 | 2 | 3 | 4 | 5 |
| 6 | Using more types of medicine leads to better effects. | 1 | 2 | 3 | 4 | 5 |
| 7 | Longer duration of medication use is better. | 1 | 2 | 3 | 4 | 5 |
| 8 | One should take medicine immediately when feeling slightly unwell. | 1 | 2 | 3 | 4 | 5 |
| 9 | Health supplements are the same as medicines. | 1 | 2 | 3 | 4 | 5 |
| 10 | Taking health supplements can reduce the dosage of medicine needed. | 1 | 2 | 3 | 4 | 5 |
| 11 | Any oral medicine can be taken with milk, coffee, or beverages. | 1 | 2 | 3 | 4 | 5 |
| 12 | Any medicines can be taken at the same time. | 1 | 2 | 3 | 4 | 5 |
| 13 | Use antibiotics to treat colds and fever. | 1 | 2 | 3 | 4 | 5 |
| 14 | Antibiotics are the same as anti-inflammatory drugs. | 1 | 2 | 3 | 4 | 5 |
| 15 | Antibiotics can kill any bacteria and viruses. | 1 | 2 | 3 | 4 | 5 |
| 16 | When people around you have a cold, fever, or other illnesses, you can take antibiotics for prevention. | 1 | 2 | 3 | 4 | 5 |
| 17 | Over-the-counter (OTC) drugs do not have any adverse effects. | 1 | 2 | 3 | 4 | 5 |
| 18 | Adverse drug reactions are considered medical accidents. | 1 | 2 | 3 | 4 | 5 |
| 19 | When adverse drug reactions occur, other medicines should be used for symptomatic relief. | 1 | 2 | 3 | 4 | 5 |
| 20 | New drugs are definitely more effective and safer than old drugs. | 1 | 2 | 3 | 4 | 5 |
| 21 | Regardless of the condition, try not to use "addictive" drugs like sleeping pills or painkillers. | 1 | 2 | 3 | 4 | 5 |
| 22 | All medicines can be stored at room temperature. | 1 | 2 | 3 | 4 | 5 |
| 23 | The storage of any medicine does not need to consider the effects of "light exposure". | 1 | 2 | 3 | 4 | 5 |
| 24 | Children (infants) can take adult medicines, just with an appropriate dose reduction. | 1 | 2 | 3 | 4 | 5 |
| 25 | Because bodily functions decline compared to younger adults, elderly people should reduce their medication doses on their own. | 1 | 2 | 3 | 4 | 5 |
| 26 | Whether male or female, any medicine can be used during pregnancy preparation without considering risks. | 1 | 2 | 3 | 4 | 5 |
| **No** | **Knowledge Yes/No Items** | **Yes** |  |  |  | **No** |
| 27 | Do you know the difference between prescription and over-the-counter (OTC) drugs? | 5 |  |  |  | 1 |
| 28 | Do you know the composition and meaning of the drug approval number? | 5 |  |  |  | 1 |
| 29 | Do you know that the dosage of some drugs needs adjustment based on your liver and kidney function? | 5 |  |  |  | 1 |
| 30 | Do you know that the dosage of the same drug for treating and preventing a disease might be different? | 5 |  |  |  | 1 |
| 31 | Do you know that some drug combinations can cause poisoning, liver/kidney damage, or other serious consequences? | 5 |  |  |  | 1 |
| 32 | Do you know how to check if a medicine has deteriorated? | 5 |  |  |  | 1 |
| **No** | **Knowledge Items (Positive)** | **Strongly Disagree** | **Disagree** | **Neutral / Don't Know** | **Agree** | **Strongly Agree** |
| 33 | Vaccination is the most effective and economical measure to prevent some diseases. | 1 | 2 | 3 | 4 | 5 |
| 34 | Pharmacists can provide good advice on whether I need to take medication. | 1 | 2 | 3 | 4 | 5 |
| 35 | Pharmacists can provide good advice on how to take my medicines correctly. | 1 | 2 | 3 | 4 | 5 |
| 36 | Family members or women who are pregnant or breastfeeding should check the "Contraindications/Special Population Precautions" section of the drug leaflet or consult a doctor or pharmacist before using any medicine. | 1 | 2 | 3 | 4 | 5 |
| **No** | **Behavior Items (Negative)** | **Always** | **Often** | **Sometimes** | **Occasionally** | **Never** |
| 37 | Purchase medicines based on your own experience or advertising. | 5 | 4 | 3 | 2 | 1 |
| 38 | When seeing a doctor, specifically ask the doctor to prescribe a certain medicine. | 5 | 4 | 3 | 2 | 1 |
| 39 | Follow the advice of salesclerks when buying medicine at a pharmacy. | 5 | 4 | 3 | 2 | 1 |
| 40 | Take Chinese herbal medicines on your own while taking Western medicines. | 5 | 4 | 3 | 2 | 1 |
| 41 | If a child refuses medicine, pinch his/her nose to force the medicine down. | 5 | 4 | 3 | 2 | 1 |
| 42 | Take medicines that have passed their expiration date. | 5 | 4 | 3 | 2 | 1 |
| 43 | Not paying attention to the time intervals between taking medicines. | 5 | 4 | 3 | 2 | 1 |
| 44 | Regardless of the dosage form, split or crush tablets before taking them. | 5 | 4 | 3 | 2 | 1 |
| 45 | Take several different medicines together without consulting a doctor or pharmacist. | 5 | 4 | 3 | 2 | 1 |
| 46 | After taking a prescription medicine for a few days and feeling no effect, change the medicine on your own. | 5 | 4 | 3 | 2 | 1 |
| 47 | To make the medicine "work faster", double the initial dose on your own. | 5 | 4 | 3 | 2 | 1 |
| 48 | To "consolidate the curative effect", continue taking the medicine beyond the required course after recovery or completion of the prescribed duration. | 5 | 4 | 3 | 2 | 1 |
| 49 | When adverse drug reactions occur, simply stop the medicine without any other action (like consulting a doctor/pharmacist or seeking help at a hospital/pharmacy). | 5 | 4 | 3 | 2 | 1 |
| **No** | **Behavior Items (Positive)** | **Never** | **Occasionally** | **Sometimes** | **Often** | **Always** |
| 50 | Use a doctor's prescription to buy prescription drugs at the pharmacy. | 1 | 2 | 3 | 4 | 5 |
| 51 | Read the drug leaflet carefully before using each medicine. | 1 | 2 | 3 | 4 | 5 |
| 52 | Check the side effects of a medicine and how they might appear before taking it. | 1 | 2 | 3 | 4 | 5 |
| 53 | Understand the contraindications (alcohol, driving, etc.) when using a particular medicine. | 1 | 2 | 3 | 4 | 5 |
| 54 | Read the instructions for using special device/delivery systems (e.g., inhalers) carefully before use. | 1 | 2 | 3 | 4 | 5 |
| 55 | Consult a pharmacist when unsure how to use a medicine (e.g., call medication consultation hotline, visit consultation center, ask window pharmacist, online consultation). | 1 | 2 | 3 | 4 | 5 |
