# Supplementary material for: Psychosocial and clinical determinants of medication adherence among elderly chronic disease patients in China
Source: Front Pharmacol. 2026 Jun 8;17:1836686. doi: 10.3389/fphar.2026.1836686 (PMC13284060; doi:10.3389/fphar.2026.1836686)
Supplement: Supplementary file 2 [file Supplementaryfile2.docx]

Supplementary Table 2 Baseline characteristics comparison: excluded versus retained participants

| **Variables** | **Total(N=1007)** | **Testdata(N=952)** | **Triandata(N=55)** | **p** |
| --- | --- | --- | --- | --- |
| BMI,Mean±SD | 25.043 ±4.622 | 25.052 ±4.667 | 24.893 ±3.803 | 0.804 |
| Number_of_chronic_diseases,Mean±SD | 2.238 ±1.168 | 2.253 ±1.170 | 1.982 ±1.114 | 0.094 |
| Number_of_medications,Mean±SD | 4.078 ±5.120 | 4.139 ±5.239 | 3.036 ±1.972 | 0.121 |
| Necessity_Beliefs,Mean±SD | 19.378 ±4.194 | 19.384 ±4.189 | 19.273 ±4.310 | 0.848 |
| Concern_Beliefs,Mean±SD | 14.364 ±4.361 | 14.346 ±4.347 | 14.691 ±4.630 | 0.568 |
| Self_efficacy_score,Mean±SD | 27.643 ±7.242 | 27.736 ±7.173 | 26.036 ±8.269 | 0.091 |
| Medication_knowledge,Mean±SD | 97.535 ±13.577 | 97.749 ±13.055 | 93.836 ±20.439 | 0.038 |
| Medication_attitude,Mean±SD | 33.447 ±6.448 | 33.392 ±6.312 | 34.400 ±8.478 | 0.26 |
| Medication_practice,Mean±SD | 69.317 ±10.618 | 69.352 ±10.644 | 68.709 ±10.232 | 0.663 |
| Adherence_group, n(%) |  |  |  | 0.971 |
| 0 | 556（55.2%） | 525（55.1%） | 31（56.4%） |  |
| 1 | 451（44.8%） | 427（44.9%） | 24（43.6%） |  |
| Gender, n(%) |  |  |  | 0.838 |
| Female | 517（51.3%） | 490（51.5%） | 27（49.1%） |  |
| Male | 490（48.7%） | 462（48.5%） | 28（50.9%） |  |
| Marital_status, n(%) |  |  |  | 0.581 |
| Married | 833（82.7%） | 787（82.7%） | 46（83.6%） |  |
| Widowed/Single/Divorced | 174（17.3%） | 165（17.3%） | 9（16.4%） |  |
| Smoking_or_not, n(%) |  |  |  | 0.112 |
| Non-smoker | 700（69.5%） | 659（69.2%） | 41（74.5%） |  |
| Smoker | 227（22.5%） | 220（23.1%） | 7（12.7%） |  |
| Former smoker | 80（7.9%） | 73（7.7%） | 7（12.7%） |  |
| Drinking_or_not, n(%) |  |  |  | 0.814 |
| Non-drinker | 715（71.0%） | 674（70.8%） | 41（74.5%） |  |
| Drinker | 49（4.9%） | 47（4.9%） | 2（3.6%） |  |
| Former drinker | 243（24.1%） | 231（24.3%） | 12（21.8%） |  |
| Employment_status, n(%) |  |  |  | 0.188 |
| Re-employed or other employment | 69（6.9%） | 64（6.7%） | 5（9.1%） |  |
| Retired at home | 793（78.7%） | 755（79.3%） | 38（69.1%） |  |
| Caring for grandchildren | 145（14.4%） | 133（14.0%） | 12（21.8%） |  |
| History_of_ADR, n(%) |  |  |  | 0.616 |
| No_ADR | 783（77.8%） | 741（77.8%） | 42（76.4%） |  |
| Yes_ADR | 224（11.3%） | 211（11.4%） | 13（9.1%） |  |
